# Supplementary material for: Adapting the comprehensive assessment of acceptance and commitment therapy processes (CompACT) questionnaire for contextual relevance in Uganda: A comprehensive approach
Source: Glob Ment Health (Camb). 2025 Jun 2;12:e57. doi: 10.1017/gmh.2025.10018 (PMC12186557; doi:10.1017/gmh.2025.10018)
Supplement: Mugarura et al. supplementary material [file S2054425125100186sup001.docx]

**CompACT (Luganda version)**

**Amannya go**……………………………………….…………………………………………………………………..

**Ennaku z’omwezi**…………………………………………………….

**Gera ebigambibwa wammanga 23 ng’okozesa olupimo luno**:

| **0** | **1** | **2** | **3** | **4** | **5** | **6** |
| --- | --- | --- | --- | --- | --- | --- |
| Ngaanira ddala | Ngaana | Ngaana mu | Sikkiriza ate sigaana | Nzikirizaamu | Nzikirizaa | Nzikiririza ddala |

| 1 | Nsobola okuzuula ebintu ebikulu mu bulamu bwange ne mbigoberera | 0 | 1 | 2 | 3 | 4 | 5 | 6 |
| --- | --- | --- | --- | --- | --- | --- | --- | --- |
| 2 | Ekimu ku biruubirirwa byange ebikulu bwe butabeera na bulumi ku mutima | 0 | 1 | 2 | 3 | 4 | 5 | 6 |
| 3 | Ebintu eby’omugaso mbikola mpapirira awatali kubissako nnyo omwoyo | 0 | 1 | 2 | 3 | 4 | 5 | 6 |
| 4 | Ng’ezaako okubeera ne bye nkola okutangira ebirowoozo ebibi oba okuwulira obubi | 0 | 1 | 2 | 3 | 4 | 5 | 6 |
| 5 | Nkola ebintu mu ngeri ekwatagana n’engeri gye nandyagadde okubeeramu mu bulamu bwange | 0 | 1 | 2 | 3 | 4 | 5 | 6 |
| 6 | Neesanga nga ntwaliddwa nnyo ebirowoozo ne sisobola kukola bintu bye njagala ennyo okukola | 0 | 1 | 2 | 3 | 4 | 5 | 6 |
| 7 | Nsalawo okusinziira ku bintu ebikulu gy’endi, ne bwe kibanga kinkalubirira | 0 | 1 | 2 | 3 | 4 | 5 | 6 |
| 8 | Neebulira nti waliwo ebirowoozo bye ssirina kubeera nabyo | 0 | 1 | 2 | 3 | 4 | 5 | 6 |
| 9 | Nzibuwalirwa nnyo okukumira ebirowoozo kukiba kiriwo mu kiseera ekyo | 0 | 1 | 2 | 3 | 4 | 5 | 6 |
| 10 | Neeyisa okusinziira kw’ebyo bye nzikiririzaamu nga nze | 0 | 1 | 2 | 3 | 4 | 5 | 6 |
| 11 | Nfuba nnyo okwewala embeera eziyinza okundeetera ebirowoozo ebibi oba okuwulira obubi | 0 | 1 | 2 | 3 | 4 | 5 | 6 |
| 12 | Ne bwe mba nga nkola ebintu ebyamakulu gyendi, neesanga mbikola sibitaddeeko mwoyo | 0 | 1 | 2 | 3 | 4 | 5 | 6 |
| 13 | Ndi mwetegefu okufuna ebirowoozo eby’engeri yonna, n’embeera zonna (ennaku, ennyike, n’ebirala), awatali kugezaako kubikyusa oba okubiremesa okujja | 0 | 1 | 2 | 3 | 4 | 5 | 6 |
| 14 | Nkola ebintu ebirina amakulu gyendi ne bwendaba nga bizibu okukola | 0 | 1 | 2 | 3 | 4 | 5 | 6 |
| 15 | Nfuba nnyo okwetangira ebirowoozo ebinzijja mumbeera | 0 | 1 | 2 | 3 | 4 | 5 | 6 |
| 16 | Neesanga bwesanzi nga nkola emirimu oba eby’okukola ebirala nga simanyi nti ndi mukubikola | 0 | 1 | 2 | 3 | 4 | 5 | 6 |
| 17 | Nsobola okugoberera enteekateeka zange ez’ekiseera ekiwanvu ne mu kiseera nga bye nkola bitambula mpola | 0 | 1 | 2 | 3 | 4 | 5 | 6 |
| 18 | Ekintu ne bwe kiba nga kikulu gyendi, sitera kukikola singa kiba nga kijja kunnyiiza | 0 | 1 | 2 | 3 | 4 | 5 | 6 |
| 19 | Kirabika ebintu byange mbikola bukozi nga ssimanyi na bulungi kiki kyenkola | 0 | 1 | 2 | 3 | 4 | 5 | 6 |
| 20 | Ebirowoozo biba birowoozo bulowoozo – tebifuga kiki kye nkola | 0 | 1 | 2 | 3 | 4 | 5 | 6 |
| 21 | Bye nzikiririzaamu byeyolekera ddala mu nneeyisa yange | 0 | 1 | 2 | 3 | 4 | 5 | 6 |
| 22 | Nsobola okutwala ebirowoozo n’embeera *(*ennaku, ennyike, n’ebirala) nga bwe bizze, awatali kugezaako okubifuga oba okubyewala | 0 | 1 | 2 | 3 | 4 | 5 | 6 |
| 23 | Nsobola okusigala nga nkola ekintu bwekiba nga kya mugaso gyendi | 0 | 1 | 2 | 3 | 4 | 5 | 6 |
